# Avian poxvirus in a free-range juvenile speckled (rock) pigeon (*Columba guinea*)

**DOI:** 10.4102/jsava.v86i1.1259

**Published:** 2015-04-30

**Authors:** Dauda G. Bwala, Folorunso O. Fasina, Neil M. Duncan

**Affiliations:** 1 Poultry Reference Centre, Department of Production Animal Studies, University of Pretoria, South Africa; 2 Department of Production Animal Studies, University of Pretoria, South Africa; 3 Pathology Section, Department of Paraclinical Sciences, University of Pretoria, South Africa

## Abstract

A flightless wild juvenile rock pigeon (*Columba guinea*) with pox-like lesions was picked up on the premises of the Faculty of Veterinary Science, University of Pretoria, Onderstepoort. The pigeon was housed overnight for possible treatment the following day but died before any other intervention could be instituted. At necropsy, coalescing masses of yellowish nodular cutaneous tumour-like lesions principally on the featherless areas were noticed on the dead pigeon’s head as well as the beak. Histological examination of the sampled skin lesions revealed multifocal areas of hypertrophic and hyperplastic epidermal epithelial cells with eosinophilic intracytoplasmic inclusion bodies (Bollinger bodies). Extract from the lesion was processed and inoculated on the chorioallantoic membranes (CAM) of 11-day-old embryonated chicken eggs and this produced pocks on one of the CAM at day 7 post-inoculation. Electron microscopy confirmed the presence of poxvirus in the CAM with the pock lesions.

## Introduction

Avian pox is a cutaneous condition in birds that is characterised by raised, wart-like lesions, mainly on the featherless portions of the skin. It is a common and well-known disease of chickens, turkeys, pet and wild birds (Tripathy 1991; Tripathy & Reed 2003). The disease is caused by the Avipoxvirus of the Poxviridae family and has been reported in at least 232 bird species from 23 orders (Bolte, Meurer & Kaleta 1999). A slowly spreading disease, avian pox is an economically important disease of chickens and turkeys as it can cause egg production decrease and even mortality, especially in commercial poultry (Tripathy & Reed 2003). In South Africa, the disease is mostly seen in poultry kept in free-range holdings (S.P.R. Bisschop [Avimune Pty Ltd, South Africa], pers. comm., 03 November 2009). The disease is spread by biting arthropods such as mosquitoes and mites (Proctor & Owens 2000), as well as through infective aerosols, contaminated feed or water, and skin trauma resulting from pecking by other birds. The disease manifests itself in both cutaneous and diphtheritic forms. The cutaneous form develops on featherless areas of the body and is characterised by epidermal discrete nodular proliferative lesions whilst the diphtheritic form is characterised by fibro-necrotic and proliferative lesions in the mouth, oesophagus and mucous membrane of the upper respiratory tract (Tripathy 1991; Tripathy & Reed 2003).

The speckled pigeon (*Columba guinea*), also known as the rock pigeon, is a member of the Columbidae family and is endemic to sub-Saharan Africa (Urban, Fry & Keith 1986), including southern Africa (Colahan 1997). It lives on mountains cliffs, rocky gorges, boulder-strewn hills, urban and rural buildings (Hockey, Dean & Ryan 2006) and feeds on a wide range of seeds, fruits and leaves (Hargreaves 1992; Little 1994; Pepler & Pepler 1991).

As speckled pigeons co-exist with other birds in the wild, as well as domesticated birds in human habitations, there is a high possibility of contact with commercial and backyard poultry, cross-transmission of pathogens and establishment of contagious diseases in domesticated species. In this study, a case of *Avipoxvirus* infection in a speckled pigeon was investigated and reported.

## Case report

A wild pigeon was picked up on the premises of the Faculty of Veterinary Science, University of Pretoria, Onderstepoort. The bird was lethargic and could only fly short distances of less than 2 m when approached. On physical examination, some parts of the head as well as the beak were covered with yellowish nodular or cutaneous tumour-like lesions, principally on the featherless areas. The pigeon was quarantined to be taken to the exotic bird clinic the following day for treatment, but died overnight.

At necropsy, the bird was aseptically opened and all organs were examined for gross lesions; however, no gross lesions were observed apart from the previously described nodular growth on the head and beak region. Samples of the nodular lesion were collected in a locally prepared antibiotic solution (benzyl penicillin sodium 10  000 IU/mL and 10 mg/mL streptomycin sulphate) for viral isolation and identification. The whole head with the remaining nodules was preserved in 10% buffered formalin for routine histopathological examination.

### Viral isolation and electron microscopy

Viral isolation was carried out on chorioallantoic membrane (CAM) of 11-day-old specific pathogen-free chicken embryos (Joklik 1962; Kotwal & Abrahams 2004). The nodular lesions collected in the antibiotic solution at necropsy were homogenised using an Ultra Turrax^®^ homogeniser and suspended in a buffered lactose peptone (BLP) solution with antibiotics. The supernatant was decanted into a centrifuge tube and spun at 400 × g for 5 min. Using a 1 mL syringe with 18 G x 1½” needle, the supernatant was transferred and double-filtered through 5.00 μm and 0.45 μm filters. Thereafter, 0.1 mL of the resulting filtrate was inoculated onto the CAM of six 11-day-old chicken embryos from a specific pathogen-free (SPF) flock (Avi-Farms, South Africa). The inoculated embryos were incubated at 37 °C for 6 days and candled daily. Eggs with dead embryos were opened and examined for pock lesions on the CAM. The filtered suspension used for egg inoculation was plated out on blood tryptose agar (BTA) and incubated at 37 °C for 48 h for a sterility check. CAMs showing pock lesions from inoculated eggs were harvested and an aliquot of this harvest was sent to the Electron Microscopy Unit of the Department of Anatomy and Physiology, University of Pretoria for electron microscopy.

### Histopathology

Fixed tissue samples in 10% buffered formalin were embedded in paraffin and sectioned at 4 μm thickness. Each section was stained with haematoxylin and eosin (H&E) (Fischer *et al.* 2008) and slides were examined under a light microscope to establish a morphological diagnosis (Bollinger 1873).

## Results

### Clinical signs, gross and microscopic examination

**FIGURE 1:**
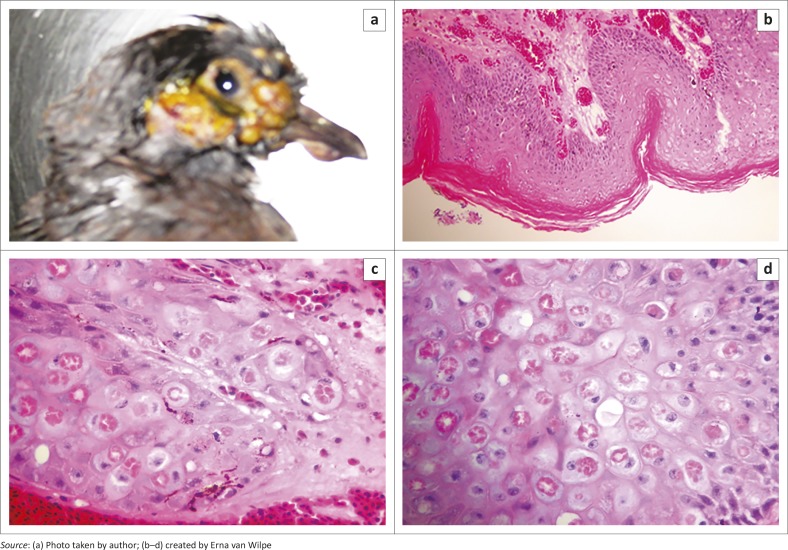
(a) Yellowish nodular cutaneous lesions on the featherless regions of the head and on the beak. (b) Keratinised epithelium revealed multifocal epidermal hyperplasia with the formation of thick rete pegs extending deep into the dermis (H&E 100X). (c) Pox-infected cells showing distended cytoplasm with eosinophilic cytoplasmic inclusion bodies (Bollinger bodies) surrounded by a pale halo. Infected cells have their nucleus compressed to the side (H&E 400X). (d) Pox-infected cells showing distended cytoplasm with eosinophilic cytoplasmic inclusion bodies (Bollinger bodies) surrounded by a pale halo. Infected cells have their nucleus compressed to the side (H&E 1000X).

*Source*: (a) Photo taken by author; (b-d) created by Erna van Wilpe

The pigeon was identified as juvenile speckled (rock) pigeon prior to submission for necropsy. Physical examination revealed superficially ulcerated parts of coalescing yellowish masses on the featherless areas of the head, the rim and base of the beak and the periocular areas. A few nodules were observed on the dorsum of the head within the feathered areas (Figures 1a and 1b).

At necropsy, no visible gross abnormality was observed but the bird presented with a prominent keel bone (as a result of emaciation) and lesions described above (Figure 1a). No diphtheritic lesions were found.

Histological examination of the H&E-stained lesions revealed keratinised epithelium with multifocal areas of epidermal hyperplasia and the formation of thick rete pegs, which extended deep into the dermis with exophytic growth that resulted in bulging of the epithelium (Figure 1b). The epidermal cells within these areas of hyperplasia were swollen but maintained their cellular outline. The cytoplasm varied from pink and finely granular to dark pink irregular aggregates with peripheral clearing within the cytoplasmic space. These cytoplasmic inclusions were usually multiple, but within some cells a single inclusion was present. The cytoplasmic inclusion bodies were eosinophilic or acidophilic (Bollinger bodies). The nuclei were compressed to one side of the cell and most of the affected cells were found within the *stratum spinosum*; in addition, the *stratum basale* showed typical changes (Figures 1c and 1d).

The outer surfaces of the hyperplastic nodules were ulcerated with necrosis and secondary invasion of the necrotic tissue by masses of small coccoid bacteria was observed. The blood vessels within the superficial dermis were dilated (hyperaemia) and many were packed with heterophils. The superficial dermis showed mild heterophilic infiltration, whilst in some areas there was a massive heterophilic infiltrate adjacent to the edges of the rete pegs within the deeper part of the dermis (Figures 1b and 1c).

### Viral isolation and electron microscopic identification

Inoculation onto BTA did not yield any bacterial growth after 48 h of incubation. Grossly, signs of viral growth on the CAM of one egg with a live embryo on day 6 post-inoculation were indicated by focal white, opaque pocks on a few spots. The other five eggs inoculated died before day 6 but had no pock lesions. The CAM with the pock lesions was harvested and an aliquot was sent for electron microscopy. Results obtained from the Electron Microscopy Unit confirmed negatively stained virus particles with typical poxvirus morphology as previously described (Catroxo *et al.* 2009; Weli *et al.* 2004).

## Discussion

Avian pox affects a wide variety of avian species, including wild and pet birds as well as commercial poultry. Most diagnoses of this proliferative disease are based on clinical signs and histopathological examination of affected organs (Bollinger 1873). In this case, diagnosis was based on clinical signs and histopathology as well as viral isolation and confirmation using electron microscopy (Bollinger 1873; Catroxo *et al.* 2009; Joklik 1962; Kotwal & Abrahams 2004; Weli *et al.* 2004). It was hypothesised that the poxvirus infection or its sequelae was the main cause of death in this rock pigeon as no other pathology was observed. The cutaneous eye and beak lesions had interfered with the bird’s ability to feed and drink water with consequent starvation, dehydration, loss of condition and associated death. The massive nature and the multiplicity of the lesions around the head and the beak, the demonstration of Bollinger bodies in formalin-fixed, paraffin-embedded section (Figures 1c and 1d), the isolation of the virus from the pock lesion and its positive identification through electron microscopy were all confirmatory of poxvirus infection. Whether the infection had a systemic component could not be confirmed in this study as the bird died overnight and no evaluation of the blood was performed. However, no lesions suggestive of the diphtheritic form of the disease were observed in the mouth, oesophagus or trachea. As no pathologic lesions were observed in any other organs, virus isolation from other organs was not attempted. In the event that the bird had compromised ingestion as suggested in this current case, there may have been reduced activity of the digestive system with possible build-up of bacteria. However, in this investigation, this possibility was not investigated. Masses of small coccoid bacteria were demonstrated in the ulcerated, hyperplastic, necrotic tissue of the nodule. The interplay of other factors including secondary bacterial infection and possible sepsis may have worsened the prognosis in this bird. The hyperaemic blood vessels within the superficial dermis and the infiltration of heterophils in the superficial dermis and tissues adjacent to the edges of the rete pegs in the deeper part of the dermis were an indication of an on-going infectious or inflammatory process. This is in agreement with previous confirmation that pox infection leads to debilitation with subsequent sepsis and fungal infection (Kim *et al.* 2003).

The course of the uncomplicated cutaneous form of avian poxvirus infection is 3–4 weeks. However, when such infection is caused by a virulent strain of avian pox, this duration may be exceeded and thus may interfere with the bird’s normal functions, resulting in significantly higher mortality. Morbidity and mortality of up to 50% have been observed in severe cases of avian poxvirus infection (Tripathy & Reed 2003). Blindness may also occur as a result of eye lesions and the resulting starvation because of inability to feed has resulted in losses in turkeys with pox infection (Tripathy & Reed 2003). We confirmed that the lethargic pigeon died of a severe case of avian poxvirus infection.

## Conclusion

Disease surveillance for poultry disease should include careful monitoring of wild birds as some of these species co-exist with other wild birds and free-range domesticated birds; in addition, they may contaminate the environment where commercial birds are raised and exchange pathogens with commercial flocks.

## References

[CIT0001] Bollinger O., 1873, ‘Ueber Epithelioma contagiosum beim Havshuhn und die sogenannten Pocken des Geflugels’ [About contagious epithelioma in poultry and the so-called avian pox], *Virchows Archiv für Pathologische Anatomie und Physiologie und für Klinische Medizin* 58, 349–361. 10.1007/BF02105254

[CIT0002] Bolte A.L., Meurer J. & Kaleta E.F., 1999, ‘Avian host spectrum of avipoxviruses’, *Avian Pathology* 28, 415–432. 10.1080/03079459994434 26911595

[CIT0003] Catroxo M.H.B., Pongiluppi T., Melo N.A., Milanelo L., Petrella S., Martins A.M.C.P.F. *et al*, 2009, ‘Identification of poxvirus under transmission electron microscopy during outbreak period in wild birds in Sao Paulo, Brazil’, *International Journal of Morphology* 27, 577–585. 10.4067/S0717-95022009000200043

[CIT0004] Colahan B.D., 1997, ‘Rock pigeon’, in Harrison J.A., Allan D.G., Underhill L.G., Herremans M., Tree A.J., Parker V. *et al.* (eds.), *The atlas of Southern African birds*, vol. 1, pp. 502–503, BirdLife International, Johannesburg.

[CIT0005] Fischer A.H., Jacobson K.A., Rose J. & Zeller R., 2008, ‘Hematoxylin and eosin staining of tissues and cell sections’, *Cold Spring Harbor Protocol*, 01 May 2008, pdb.prot4986, Woodbury, New York. 10.1101/pdb.prot4986 21356829

[CIT0006] Hargreaves B.J., 1992, ‘A spurge *Jatropha zeyheri* eaten by rock pigeon (*Columba guinea*), Botswana’, *Babbler* 23, 43–45.

[CIT0007] Hockey P.A.R., Dean W.R.J. & Ryan P.G. (eds.), 2006, *Roberts birds of South Africa*, 7th edn., The Trustees of the John Voelcker Bird Book Fund, Cape Town.

[CIT0008] Joklik W.K., 1962, ‘The purification of four strains of poxvirus’, *Virology* 18, 9–18. 10.1016/0042-6822(62)90172-1 14036977

[CIT0009] Kim T.J., Schnitzlein W.M., Mcaloose D., Pessier A.P. & Tripathy D.N., 2003, ‘Characterization of an avianpox virus isolated from an Andean condor (*Vultur gryphus*)’, *Veterinary Microbiology* 96, 237–246. 10.1016/j.vetmic.2003.08.003 14559171

[CIT0010] Kotwal G.J. & Abrahams M., 2004, ‘Growing poxviruses and determining virus titer’, in Isaacs S.N. & Totowa N.J. (eds.), *Vaccinia virus and poxvirology: Methods in molecular biology*, pp. 101–112, Humana Press, New Jersey 10.1385/1-59259-789-0:101 15114010

[CIT0011] Little R.M., 1994, ‘Marked dietary differences between sympatric feral rock doves and rock pigeons’, *South African Journal of Zoology* 29, 33–35.

[CIT0012] Pepler D. & Pepler C., 1991, ‘Food of rock pigeon in the Karoo’, *Promerops* 199, 9–10.

[CIT0013] Proctor H. & Owens I., 2000, ‘Mites and birds: Diversity, parasitism, and coevolution’, *Trends in Ecology and Evolution* 15, 358–364. 10.1016/S0169-5347(00)01924-8 10931667

[CIT0014] Tripathy D.N. & Reed W.M., 2003, ‘Pox’, in Saif Y.M., Barnes H.J., Glisson J.R., Fadly A.M., McDougald L.R. & Swayne D.E. (eds.), *Diseases of poultry*, 11th edn., pp. 253–269, Iowa State University Press, Ames.

[CIT0015] Tripathy D.N., 1991, ‘Pox’, in Calnek B.W., Barnes H.J., Beard C.W., Reid W.M. & Yoder Jnr H.W.. (eds.), *Diseases of poultry*, pp. 583–596, Iowa State University Press, Ames.

[CIT0016] Urban E.K., Fry C.H. & Keith S. (eds.), 1986, *The birds of Africa*, vol. 2, Academic Press, London.

[CIT0017] Weli S.C., Okeke M.I., Tryland M., Nilssen O. & Traavik T., 2004, ‘Characterization of avipoxviruses from wild birds in Norway’, *Canadian Journal of Veterinary Research* 68, 140–145. 15188959 PMC1142158

